# Exploring Residents’ Purchase Intention of Green Housings in China: An Extended Perspective of Perceived Value

**DOI:** 10.3390/ijerph18084074

**Published:** 2021-04-13

**Authors:** Shiwen Zhao, Liwen Chen

**Affiliations:** School of Economics and Management, Hebei University of Technology, Tianjin 300401, China; coastline7@126.com

**Keywords:** green housing, perceived value, residents’ perception, personal trait, purchase intention

## Abstract

The promotion of green housings (GHs) is considered a potentially effective way to save energy, reduce air pollution, and promote industrial upgrading. However, the low level of public acceptance of GHs leads to insufficient GH market penetration in China. Thus, it is significant to explore GH purchase decisions to understand and enhance the market demand for GHs effectively. From an extended perspective of perceived value, this study proposes a comprehensive research model that integrates residents’ perceptions and personal traits to examine the influencing mechanism of residents’ intention to purchase GHs. The proposed model is empirically tested using data collected from 728 urban residents in China. The results reveal that perceived value is a crucial predictor of GH purchase intention. All dimensions of perceived benefits—including perceived functional benefits, perceived emotional benefits, perceived green benefits, and perceived social benefits—have a positive influence on perceived value, while perceived performance risks have a negative influence on perceived value. Two types of personal traits, namely, environmental concern and social trust, significantly affect residents’ perceived benefits and perceived risks. The findings contribute to a more in-depth analysis of the effects of residents’ perceptions and personal traits on GH purchase behavior. Furthermore, suggestions for policymakers and developers on popularizing GHs are proposed.

## 1. Introduction

The development of the construction industry is accompanied by large amounts of resource and energy consumption, which results in a series of environmental problems, such as the greenhouse effect and extreme weather disasters. In developing countries, the building industry accounts for approximately 40% of total energy consumption and 30% of total greenhouse gas emissions [1]. This is especially true for China due to its rapid urbanization; if the development model of the construction industry does not change, then the industry’s adverse effects on the environment will be further aggravated. Green buildings (GBs), which are emerging as a new architectural concept in response to sustainable development, are expected to be one of the promising methods for tackling environmental and energy issues [2,3]. Compared to traditional buildings, GBs can promote the efficient use of energy, water, materials, and other natural resources; reduce environmental pollution; be more cost-effective within the whole building life cycle; and improve human health and well-being [4,5,6]. Fundamentally, the development of GBs is beneficial to the economy, environment, and society [7]. China’s GB definition includes residential buildings and public buildings. As a subset of GBs, green housings (GHs) can meet the general requirements of GB standards and emphasize the improvement in the quality of life of residents [8]. Since residential buildings comprise approximately two-thirds of all buildings in China, the promotion of GHs is particularly critical for the green transformation of China’s construction industry [9].

The Chinese government has been strenuously promoting GB development by implementing laws and regulations, establishing evaluation standards, and providing incentives [10]. For instance, the Ministry of Housing and Urban-Rural Development (MOHURD) of China issued the Evaluation Standards for Green Buildings in 2006 and passed updated versions in 2014 and 2019. After several important adjustments, the criteria of the latest version cover five categories, namely, safety and durability, health and comfort, convenience of life, resource conservation, and the livability of the environment. This is a low-cost and effective form of providing official information to stakeholders, which is conducive to dealing with the information asymmetry in the GH market. It is worth noting that mandated regulations and disciplines rather than incentives are more commonly adopted in China. In addition, incentive policies are mainly concentrated on the supply-side of the GH market rather than the demand side. The Chinese government issued the Green Building Action Plan in July 2020, which set the goal that by 2022, the proportion of new urban buildings in urban green buildings will exceed 70%; this goal seems ambitious and challenging. Driven by the aforementioned measures, GBs and GHs have made some progress in China. However, GHs accounted for less than 0.4% of China’s total buildings until 2017, which means that GH market development remains sluggish [8,11].

The paradox is that the Chinese government regards GHs as the dominant tendency of housing transformation, but the majority of homebuyers remain indifferent to this new eco-friendly housing. The reasons may be as follows. First, the government is mainly concerned with the role of macroeconomic regulation and control policy, but it ignores the importance of public perception and market acceptance. The possible result is that whether GHs meet the evaluation standards is overemphasized while the real needs of residents are neglected. In particular, there may be differences between the evaluation standards and residents’ expectations [12]. Second, residents may have some misunderstandings and concerns about GHs. Due to residents’ relatively limited experience or knowledge, most of them are generally unfamiliar with the main advantages of GHs or regard GHs as houses that are only beneficial to the environment. This also implies that green certification still plays a limited role in China’s residential market and only leaves vague impressions on residents. Moreover, GHs face a new set of challenges regarding price, safety, performance, maintenance, and regulation issues, which exacerbates residents’ concerns [13,14,15]. As the final purchasers of GHs, the acceptance of residents is the critical prerequisite to market demand. Therefore, the low level of awareness and acceptance of residents directly reduces developers’ motivation to adopt green practices and inhibits the great efforts made by the Chinese government [16,17]. To solve this paradox, it is of far-reaching significance to study Chinese urban residents’ GH purchase decisions.

Many scholars have explored GH purchase decisions, and many of these scholars have concentrated on the instrumental attributes of GHs [8,17,18,19,20,21,22]. The focus of these studies has been to estimate homebuyer preferences for product attributes in GH purchasing decisions. The findings have demonstrated that instrumental attributes, such as indoor air quality, insulation, temperature, humidity, ventilation, lighting, building materials, water savings, energy efficiency, levels of pollution, price, maintenance costs, etc., have heterogeneous effects on the willingness to pay for GHs. However, the success of the GH market depends on how well residents accept GHs. In other words, the motivation to purchase GHs is based on residents’ perceptions rather than GHs’ instrumental attributes [23,24]. Moreover, only focusing on the tool attributes in the evaluation system without paying attention to the actual perception of residents may not achieve good sustainability outcomes [12]. The other research perspective considers GHs as green innovation products and focuses on depicting the intrinsic motivation and process of GH purchase decision making. Related studies have investigated the effects of green values and beliefs, environmental attitude and responsibility, moral norms, and other cognitive and psychological factors on intention to purchase GHs. Certain individual-level theories and models, such as the theory of planned behavior, the values–beliefs–norms theory, the technology acceptance model, and the norm activation model, are often exploited [16,25,26,27,28,29,30]. In accordance with the theory of customer perceived value, consumer purchase decisions depend on the entire evaluation of the utility of products [31], that is, perceived value, which is obtained by thoroughly weighing perceived gains (i.e., benefits) and losses (i.e., risks) [32,33]. However, to the best of our knowledge, previous research has not expounded well on the conception of “perceived value” in the context of GHs or its role in predicting GH purchase intention. Thus, this study proposes a research framework from an extended perspective of perceived value to explore GH purchase decision-making.

It is noteworthy that residents’ perception consists of both motivations (positive) and hindrances (negative) involving multiple aspects. That is, the promotion of GHs should fully consider residents’ tradeoff between multi-dimensional perceived benefits and perceived risks [34,35]. Homebuyers consider GHs’ primary benefits to be their functional benefits with regard to comfort, health, and economy [8,12,17,34]; their emotional benefits as related to psychological satisfaction [12,36]; their green benefits associated with reduced environmental pollution and efficient energy utilization [8,21,22,37]; and their social benefits concerning individual image and social acceptance [12,26,27]. The main hindrances are perceived to be the performance risk involving uncertain stability, safety, and reliability [4,35,38,39] and the financial risk in terms of high acquisition costs, operating costs, and maintenance costs [4,28,35,38]. Both perceived benefits and perceived risks are essential factors influencing residents’ purchase intention of GHs. However, few studies have considered both positive and negative perceptions and explored their multiple dimensions. To address this gap, the current research framework was developed from an extended perspective of perceived value, allowing us to explore the multi-dimensional perception that includes what is received and what is given when homebuyers consider purchasing GHs.

Furthermore, situational factors, such as personal traits, will impact the formation of individual perceptions of products or services [40,41]. Personal traits have been frequently considered as important factors explaining individual perceptions and acceptance of green products. Since GHs are environmentally friendly products, the purchase of GHs could be regarded as a specific pro-environmental behavior. Thus, individual environmental concerns may have an effect on residents’ perception and acceptance of GHs. Additionally, personal social trust also plays an important part in the GH purchase decision. Given GHs have not gained sufficient popularity in China, trust in responsible organizations for GHs can convince the public of the benefits of GHs and weaken the public’s risk perception. Accumulating evidence has indicated that environmental concerns and social trust indirectly affect consumer purchase decisions through perception factors [8,16,28]. Thus, environmental concern and social trust, as two types of personal traits, are used as significant antecedents of residents’ perception and their intention to purchase GHs in this study.

Considering the existing research status, from an extended perspective of perceived value, this study will develop a research model that considers residents’ perception and personal traits as antecedents to explore the influencing mechanism of GH purchase intention. The study aims to make contributions in three aspects. First, as a psychological link between products and consumers, perceived value provides a new perspective for the analysis of GH purchase decisions. Second, we classify the perceived benefits and perceived risks of GHs into multiple dimensions to compare their importance, which facilitates a more accurate and targeted prediction of GH purchase behavior. Finally, we explore the influence of personal traits, including social trust and environmental concern, on residents’ perception, thereby contributing to a more in-depth understanding of the influence of personal psychological traits on GH acceptance.

The remainder of this paper is organized as follows. Section 2 proposes the related hypotheses and a research framework. Then, we introduce the research methods in Section 3. Section 4 presents the data analysis and results. We discuss the results and indicate the practical significance implications in Section 5. The final section presents the conclusion and limitations of this study.

## 2. Theoretical Framework and Research Hypotheses

### 2.1. Perceived Value

According to a widely accepted definition in previous research [32], perceived value of GHs is defined as “the resident’s overall perception of GHs based on his or her tradeoff between benefits and sacrifices”. As has been found in a range of different research backgrounds, a higher perception of value often results in a more positive purchase decision. In the context of green consumption behavior, Park and Kwon revealed that users’ perceived value of energy-saving behavior positively affects their behavioral intention [42]. Kim et al. indicated that consumers’ intention to adopt electric vehicles is positively determined by their overall value perception [43]. Wang et al. suggested that individuals’ value perceptions of ridesharing services positively affect their intention to use ridesharing services [44]. In light of the abovementioned literature, the effect of perceived value on residents’ purchase intention of GHs is assumed as follows:

**Hypotheses** **1** **(H1).** *Perceived Value Positively Affects GH Purchase Intention*.

### 2.2. Perceived Benefit

Perceived functional benefits refer to residents’ perception of the utilitarian, functional, and physical aspects of GHs [45]. The functional benefits are derived from the instrumental characteristics or attributes of GHs, mainly including comfort, health, and economy. When considering the composition of the perceived benefits of GHs, researchers usually include “perceived usefulness” as the key element corresponding to functional value [16,28]. According to previous research results, GHs can provide residents with a more comfortable and safer indoor environment, improve their health and productivity, and save water and electricity costs [2,46,47]. Hu et al. suggested that performance considerations, such as health and comfort, are the key motivations for attracting consumers to purchase GHs [17]. Liu et al. found in a survey conducted in Taiwan that a higher perception of the quality and economic characteristics of GHs may lead to a higher willingness to pay for residents [48]. Thus, if residents perceive higher functional benefits of GHs, then they will have a more positive overall perceived value of GHs.

Perceived emotional benefits refer to residents’ perception of the utility derived from GHs’ ability to evoke residents’ sentiment or affective states [49]. When consumers consider purchasing products or services, in addition to the attainment of performance, a good emotional experience is also the intrinsic benefit they primarily desire [45]. In other words, satiating psychological needs may also motivate customers’ purchasing behavior [50]. Corresponding to GHs, psychological needs imply a sense of reassurance, happiness, and pleasure from a better living experience in GHs than traditional housing [36]. This positive emotional expectation is conducive to residents’ better evaluation of GHs. Furthermore, as a type of altruistic behavior, purchasing GHs will make residents feel moral satisfaction by doing good deeds and then feel the intrinsic warm glow [51]. The environmentally friendly features of GHs and the pleasure and happiness from the living experience in GHs may satisfy different residents’ emotional states. When residents are more emotionally motivated, their overall perceived value of GHs may be higher.

Perceived green benefits represent residents’ perception of the effects of energy conservation and environmental protection from purchasing and living in GHs [52]. With the aggravation of global environmental issues, consumers show solicitude for the environmental attributes of products, which may impact their preference. Compared to traditional housing, GHs can reduce environmental pollution and make effective use of resources and energy throughout the life cycle [53]. Therefore, GHs have unique environmental benefits compared to traditional housing, which form residents’ perception of green value [36,54]. In particular, environmentalists will be more likely to accept GHs since they consider GH purchase behavior to be an approach for preserving the eco-environment. Chau et al. found that residents have strong preferences for the environmentally friendly characteristic of GHs and are willing to pay more for them [18]. Li et al. suggested that consumers’ perception of the environmental benefits of GHs, as the antecedent of perceived value, has an important influence on their purchase decisions [35]. Thus, residents’ perceived green benefits of GHs may produce a positive effect on overall perceived value.

Perceived social benefits refer to residents’ perception of the utility derived from GHs’ ability to enhance social self-concept, such as self-image improvement and approval of others [49]. Due to the pro-environmental attributes of GHs, the purchase of GHs may be conducive to shaping residents’ green consumer identity or positive images [55], especially because housing purchases involve a high level of public visibility [27]. In addition, by answering a call for a low-carbon lifestyle and showing the individuals’ social responsibility, residents who live in GHs are likely to obtain the goodwill of surrounding people and gain social recognition. Previous research has found that prestige seeking is a considerable motivation for purchasing GHs [12,20,37]. Jia et al. found that consumers who attach importance to a positive image are more willing to pay for energy conservation housing [26]. Zhang et al. suggested that perceived social benefits is an important antecedent of the perceived value of GHs [36]. Therefore, the more residents believe that GHs contribute to improving personal image and winning plaudits, the more value they likely perceive.

Based on the above analysis, the following assumptions are proposed:

**Hypotheses** **2a** **(H2a).** *Perceived functional benefits positively affect the perceived value of GHs*.

**Hypotheses** **2b** **(H2b).** *Perceived emotional benefits positively affect the perceived value of GHs*.

**Hypotheses** **2c** **(H2c).** *Perceived green benefits positively affect the perceived value of GHs*.

**Hypotheses** **2d** **(H2d).** *Perceived social benefits positively affect the perceived value of GHs*.

### 2.3. Perceived Risk

Perceived performance risks are defined as the probability that GHs fail to function as designed or publicized and thus cannot realize expected benefits. Since the GH market is still in the initial stage, the immaturity of the new technology and equipment used in GHs may make residents worry about its safety and reliability [15]. The presale system of China’s real estate market will lead to information asymmetry, which may provide developers with opportunities to exaggerate the performance of GHs. As it is difficult for homebuyers to intuitively understand all the performance characteristics of GHs before living in them [39], the presale process may cause residents to be anxious about the authenticity of the advertisements and promotions. Furthermore, there is a mismatch between high design standards and poor operational performance as a result of inadequate management in the operational phase in China [14,56]. This means that GHs may not maintain satisfactory performance continuously over the entire life cycle. The current literature indicates that perceived performance risks negatively affect homebuyers’ attitude and intention to purchase GHs [4,35,38]. Therefore, when residents perceive higher performance risks, they have a more negative overall perceived value of GHs.

Perceived financial risks refer to residents’ perception of the possibility of pecuniary losses caused by purchasing or maintaining GHs. Since GH providers fail to achieve economies of scale in the infancy stage, the price of GHs is generally higher than that of traditional housing. Previous studies have shown that high acquisition costs are a major roadblock to public acceptance of GHs [26,57]. Since some mechanical and electrical equipment may be used in GHs, in order to ensure the normal operations of buildings, a certain amount of money will inevitably be invested in this process. Additionally, due to the lack of a comprehensive management system and professional technical personnel, the poor operation of GHs may lead to financial uncertainty. These factors may cause residents to bear unexpected maintenance costs and other complementary expenditures in the operating stage. Extant studies have shown that the perceived financial risks formed by acquisition costs and maintenance costs negatively affect homebuyers’ purchase decisions for GHs [4,28,35]. Thus, when residents believe that GHs are more likely to cause economic losses, their overall perceived value is lower.

Based on the above analysis, the following assumptions are proposed:

**Hypotheses** **3a** **(H3a).** *Perceived performance risks negatively affect the perceived value of GHs*.

**Hypotheses** **3b** **(H3b).** *Perceived financial risks negatively affect the perceived value of GHs*.

### 2.4. Environmental Concern

Environmental concern is defined as the extent of personal attention to environmental issues and their readiness to solve them [58]. Consumers who are more concerned about environmental issues will take notice of the environmental impacts of their consumption [59]. Furthermore, they are more likely to take measures to reduce their ecological footprint by changing their original lifestyles. Many studies have indicated that environmental concern serves as a critical influencing factor of environmentally friendly behavior, such as garbage reduction [60], water conservation [61], and green purchase behavior [62]. As GHs can be considered special pro-environmental products, environmental concern has been proven to become an inherent psychological factor influencing residents’ GH purchase decisions [63].

In addition, residents with high environmental concerns are more inclined to assess the environmental performance of products. These people are more willing to pay attention to the environmental attributes of products, are sensitive to eco-friendly products, and easily perceive green value from eco-friendly products. Furthermore, as new environmentally-friendly housing, GHs may more easily satisfy environmentalists’ psychological demand and reflect their green consumer identity [64]. Furthermore, individuals passionate about environmental protection may have a higher tolerance for economic risks than others because they tend to spend additional money on pro-environmental products [41]. Jiang and Kim confirmed that environmental concerns can increase consumers’ perceived benefits and reduce their perceived costs when considering choosing green hotels [40]. In view of the aforementioned statements, residents with high environmental concerns possibly believe that GHs would be a developmental tendency of housing displacing traditional housing. Therefore, they would easily perceive the green benefits and related psychological benefits of GHs and possess a higher level of tolerance towards potential financial risk.

Based on the above analysis, the following assumptions are proposed:

**Hypotheses** **4a** **(H4a).** *Environmental concern positively affects the perceived emotional benefits*.

**Hypotheses** **4b** **(H4b).** *Environmental concern positively affects the perceived green benefits*.

**Hypotheses** **4c** **(H4c).** *Environmental concern positively affects the perceived social benefits*.

**Hypotheses** **4d** **(H4d).** *Environmental concern negatively affects the perceived financial risks*.

### 2.5. Social Trust

Rousseau et al. defined trust as “a mental condition that includes the intention to accept vulnerability in view of positive anticipations of the intentions or behavior of others” [65]. Social trust refers to the tendency of individuals to be willing to depend on the related agencies in charge of making decisions and implementing measures for public health and safety affairs [66]. In general, since most people are deficient in their professional knowledge or practical experience to appraise a new science or technology (e.g., GHs and other green products), they are more likely to rely on experts or authorities [39]. Stern proposed that people choose to invest in new energy because of trust in institutions and used the residential building energy-saving renovation project launched by the local government of Minnesota as an example to demonstrate the influence of trust on the willingness of households to participate [67]. As previous studies have shown, it is essential to consider the role of social trust in purchase decision-making for GHs since GHs are typical experiential products [16,28].

Additionally, social trust may affect how consumers perceive the product and thus play a role in determining the risk–return judgment [68]. Many previous studies have claimed that social trust can positively impact perceived benefits and negatively impact perceived risks in the study of the intention to employ particular technologies, such as renewable energy [69], electromagnetic fields [70], and electric vehicles [71]. Accordingly, residents with a higher level of social trust are more convinced of the truthfulness of the advantages of GHs and thus perceive more tangible and intangible benefits from purchasing and living in GHs. In addition, these residents may have lower concerns about uncertainty when choosing GHs.

Based on the above analysis, the following assumptions are proposed:

**Hypotheses** **5a** **(H5a).** *Social trust positively affects the perceived functional benefits*.

**Hypotheses** **5b** **(H5b).** *Social trust positively affects the perceived emotional benefits*.

**Hypotheses** **5c** **(H5c).** *Social trust positively affects the perceived green benefits*.

**Hypotheses** **5d** **(H5d).** *Social trust positively affects the perceived social benefits*.

**Hypotheses** **5e** **(H5e).** *Social trust negatively affects the perceived performance risks*.

**Hypotheses** **5f** **(H5f).** *Social trust negatively affects the perceived financial risks*.

**Figure 1:**
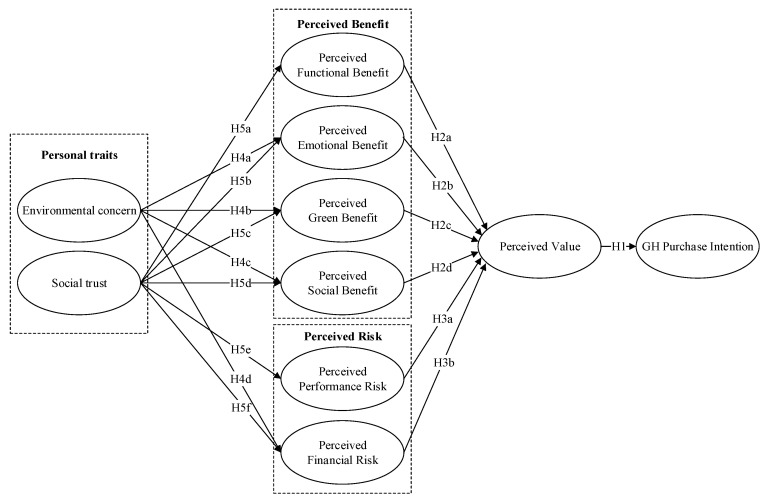
Research model of green house (GH) purchase intention.

Based on the above analysis, the research model is depicted in Figure 1.

## 3. Research Method

### 3.1. Measurement Development

The measurement items of the constructs were adopted from previous studies and were modified and adapted to the present research setting. Items for GH purchase intention were adapted from Chen et al. [72]. Items for perceived value were adapted from Kim et al. [43]. Items for perceived functional benefits were adapted from Sweeney and Soutar [49], Liu et al. [16], and Zhang et al. [36]. Items for perceived green benefits were derived from He et al. [41] and Zhang et al. [54]. Items for perceived emotional benefits and social benefits were adapted from Sweeney and Soutar [49], Zhang et al. [36], and Zhang et al. [54]. Items for perceived performance risks were adapted from Grewal et al. [73] and Featherman and Pavlou [74]. Items for perceived financial risks were adapted from Grewal et al. [73] and Li et al. [4]. Items for environmental concern were adopted from Goh and Balaji [62]. Items for social trust were adopted from Liu et al. [16]. These items are shown in Table 1. Each item is measured by a 7-Likert scale (1 = strongly disagree to 7 = strongly agree).

### 3.2. Data Collection

The survey participants mainly included urban residents of five cities in eastern China: Beijing, Shanghai, Tianjin, Shenzhen in Guangdong Province, and Suzhou in Jiangsu Province. Previous research has indicated that China’s GB development level has a high level of spatial imbalance [9]. There are five provinces with higher GB development levels than others, including Shanghai, Tianjin, Jiangsu, Beijing, and Guangdong [75]. Among these provinces, Beijing, Shanghai, and Tianjin are municipalities directly under the Central Government of China that have strong political and economic status. In addition, Suzhou and Shenzhen are the representative cities of Jiangsu and Guangdong, respectively, which have higher GB sales than other cities in the province. Thus, the regional selection in this study includes the top-ranked cities in terms of GB development level, indicating that urban residents in these areas have relatively more knowledge and experience of GHs. In addition, all five cities belong to China’s first-tier cities or new first-tier cities, which represent regions with high levels of economic development, population density, and construction scale in China. Therefore, it is reasonable and representative to explore these residents’ perceptions and purchase intentions for GHs. It is of practical significance for the gradual improvement in residents’ acceptance of GHs in various regions of China in the future.

Questionnaires were distributed online through a professional online survey platform called Wenjuanxing (www.sojump.com) from August to November 2020. Participants who completed the questionnaire effectively were paid accordingly. After eliminating those responses that were invalid due to evident logical errors or completion in less time, a final total of 728 valid questionnaires were retained. The demographic data are shown in Table 2. In terms of gender distribution, the proportion of males (50.4%) and females (49.6%) in the sample was basically the same. The main age group was 18–49 years old, with the largest number of people in the 30–39 age group. Concerning educational background, the majority of the respondents (57.4%) held a bachelor’s degree. Regarding annual household income, most of the respondents (72.6%) had an annual household income of 100–500 thousand RMB.

## 4. Data Analysis and Results

Compared with co-variance-based structural equation modeling (CB-SEM), partial least squares structural equation modeling (PLS-SEM) is an appropriate approach for explorative or extensive research models [76,77]. Thus, PLS-SEM was utilized to examine the research model in this study. We first assessed the reliability and validity of the measurement model and then examined the structural model to test the research hypotheses.

### 4.1. Measurement Model

Confirmatory factor analysis (CFA) was performed to assess the reliability and validity of the measurement model. The reliability was evaluated using the composite reliability (CR) values and Cronbach’s α values [78]. Table 3 shows that the Cronbach’s α values and the CR values of all constructs were more than 0.70, implying that the reliability was acceptable. The average variance extracted (AVE) of the constructs and standard loadings of indicators were used to test the convergent validity [79]. As shown in Table 3, all the AVEs exceeded the suggested lowest value of 0.5. Moreover, all standard loadings of items were above the benchmark value of 0.7. The discriminant validity can be evaluated by comparing the square root of the AVE with interconstruct correlations [78]. Table 4 shows that the square root of the AVE (i.e., on the diagonal) for each construct was larger than its correlations with other constructs, which suggests an acceptable level of discriminant validity [78]. As the data were self-reported and collected from a single source, Harman’s one-factor test was used to assess Common Method Variance (CMV) [80]. The results indicated that multiple factors were extracted, and the first factor explained 33.98% of the variance, implying that method variance was not an issue in this study.

### 4.2. Structural Model

**Figure 2:**
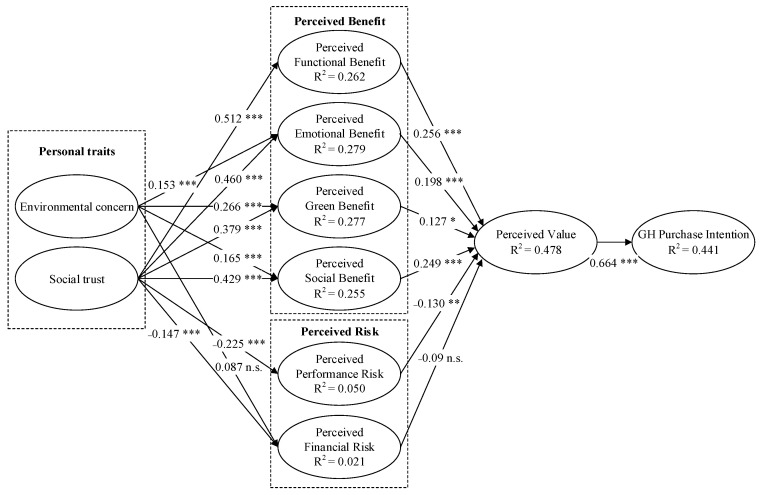
Results of hypothesis testing. Note: * *p* < 0.05; ** *p* < 0.01; *** *p* < 0.001, n.s. nonsignificant.

The PLS-SEM analysis results of the structural model are presented in Figure 2 and Table 5. As illustrated in Figure 2, the total variance of GH purchase intention explained by residents’ perception and personal traits was 44.1%, indicating adequate predictive validity [81]. Additionally, the value of Goodness of Fit (GoF) was 0.452, which confirms that it was large enough to support the global model validity. To assess the significance of the coefficient for every path proposed in the research model, a bootstrapping technique was performed with 5000 re-samples. Based on the results presented in Table 5, all hypotheses were supported except for H3b and H4d.

In detail, perceived value (PV) was found to affect Purchase intention (PI) both significantly and positively (β = 0.664, *p* < 0.001), thus supporting H1. In terms of benefits, perceived functional benefit (PFB) (β = 0.256, *p* < 0.001), perceived emotional benefit (PEB) (β = 0.198, *p* < 0.001), perceived green benefit (PGB) (β = 0.127, *p* < 0.05), and perceived social benefit (PSB) (β = 0.249, *p* < 0.001) had significant positive impacts on PV, thereby supporting H2a, H2b, H2c and H2d, respectively. In terms of risks, perceived performance risk (PPR) significantly negatively affected PV (β = −0.130, *p* < 0.01), thus supporting H3a. However, the relationship between perceived financial risk (PFR) and PV was not significant (β = −0.090, *p* > 0.05) and thus did not support H3b.

With regard to personal traits, environmental concern (EC) was found to have significant positive impacts on PEB (β = 0.153, *p* < 0.001), PGB (β = 0.266, *p* < 0.001), and PSB (β = 0.165, *p* < 0.001), thus supporting H4a, H4b and H4c, respectively. The relationship between EC and PFR was not significant (β = 0.087, *p* > 0.05) and thus did not support H4d. social trust (ST) had significant positive impacts on PFB (β = 0.512, *p* < 0.001), PEB (β = 0.460, *p* < 0.001), PGB (β = 0.379, *p* < 0.001), and PSB (β = 0.429, *p* < 0.001) and had significant negative impacts on PPR (β = −0.225, *p* < 0.001) and PFR (β = −0.147, *p* < 0.001), thus supporting H5a, H5b, H5c, H5d, H5e and H5f, respectively.

### 4.3. Effect Analysis

To gain insight into the critical motivations and hindrances of the GH purchase decision, this study calculated the total effects (the sum of the direct and indirect effects) of the constructs on the GH purchase intention. As shown in Table 6, PV was the most important motivation of PI (0.664). Additionally, PFB, PEB, PSB, and ST were relatively evident factors in determining PI, and their total effects were 0.170, 0.131, 0.165, and 0.178, respectively.

## 5. Discussion and Implications

### 5.1. Discussion

The popularization of GHs is of profound significance for achieving sustainable economic, social, and environmental development. Residents are the most important market stakeholders, and their acceptance and purchase decision of GHs has gradually attracted attention. Nevertheless, few empirical studies have explored residents’ multi-dimensional perception of GHs from the perspective of individual perception and considered the effects of individual psychological characteristics on the formation of perception. To address this gap, this study investigated the impact mechanism of residents’ intention to purchase GHs with a comprehensive research framework incorporating individual perception and psychological traits. The key findings are discussed below.

First, the overall value perception was found to have a significant positive influence on residents’ intention to purchase GHs (H1), conforming to the well-acknowledged assumption that perceived value is the crucial prerequisite for consumers to select new products or services [32,82]. Consequently, to promote residents’ acceptance and purchase of GHs, it is necessary to ensure that residents have a more positive overall evaluation of GHs, that is, a higher value perception. In particular, since the purchase costs of GHs are relatively high, residents will definitely experience a process of weighing and considering before making a final decision.

Second, the results indicate that all dimensions of perceived benefits have positive and significant effects on the value perception of GHs, and the degree of the effects are different. This result coincides with the findings of Wang et al. [83], wherein green consumption behavior is a complicated decision-making process involving multi-dimensional drivers. Specifically, perceived functional benefits were found to have the strongest effect on residents’ perceived value (H2a), confirming the findings of previous research that comfort, health, and monetary savings are the GH benefits most desired by residents [8,17]. In addition to utilitarian terms, perceived emotional benefits were found to have positive effects on residents’ perceived value of GHs (H2b), thereby supporting the results of previous studies that an overall better mood and level of happiness is also an important motivation to purchase GHs [12,36]. Coinciding with the conclusions of previous literature that social attributes are significant for consumers to purchase green products [55,84], this study found that perceived social benefits act as an essential determinant of GH purchase decisions (H2d). Although the effect of perceived green benefits on residents’ perceived value of GHs was significant (H2c), the effect was the smallest among all types of perceived benefits. This result might be because Chinese consumers tend to believe that they have no obligation to solve environmental and energy problems [85]. Therefore, even if residents know about the environmental friendliness of GHs, they probably feel that the environmental benefits of GHs are less attractive to them when determining whether to purchase GHs. Furthermore, we can further deduce that egoistic motivation outbalances altruistic motivation in the housing purchase decisions of Chinese residents. Since environmental attributes are not the main concern of residents, they must be coordinated with other private attributes in order to maximize the benefits of GHs [86].

Third, perceived performance risks and perceived financial risks were found to have different effects on residents’ perceived value of GHs. The former negatively influenced residents’ perceived value of GHs (H3a), supporting the results of previous studies [4,35]. This result implies that residents are concerned that the performance of GHs is not as good as advertised or designed and thus cannot meet their expectations. In addition, imperfect operations provided by property management companies might result in accidents and failures of GHs, which also lead to residents’ apprehension. Contrary to expectations, the effect of perceived financial risks on residents’ perceived value of GHs was insignificant (H3b). The possible reasons might be explained as follows. Since the survey areas in this study were all China’s first-tier cities or new first-tier cities where housing prices are much higher than other areas, the incremental costs of GHs appear to be a relatively low proportion of the total purchase costs. Thus, local residents may be less sensitive to the incremental costs of GHs and more tolerant of the perceived financial risks. This result is consistent with the findings of Li et al. [4], which indicated that concerns regarding technological performance remain a key barrier, while economic risks are not a major obstacle to widespread GH diffusion in China’s first-tier cities or new first-tier cities. Similar to the conclusion of Li et al. [4], Liu and Hu found that price is not the main factor causing the negative sentiment of the Chinese public towards vertical greening houses [15]. Moreover, the impact of perceived benefits among all dimensions is also relatively stronger than that of perceived risks. This finding shows that residents are more concerned with the benefits of living in GHs than with the risks.

Finally, two types of personal traits had important influences on residents’ perceptions. In agreement with the findings of previous studies [40,87], environmental concern not only increased perceived green benefits (H4b) but also increased perceived emotional benefits (H4a) and perceived social benefits (H4c). The probable reason for this result is that residents concerned about the environment could easily notice the environmental benefits of green products and envision related invisible benefits when living in GHs. The effect of environmental concern on residents’ perceived financial risks of GHs was insignificant (H4d). This implies that residents’ environmental awareness cannot be transformed into the willingness to pay more for GHs. Moreover, social trust could increase all types of perceived benefits (H5a, H5b, H5c, and H5d) and reduce both perceived risks (H5e and H5f), supporting the findings of previous research that social trust is a significant antecedent of individual perception of new technology or product [68]. Only when residents have a high degree of social trust can they be more aware of the benefits of GHs and reduce their concerns about the uncertainty. The results also agree with the view of Liu et al. [16], which indicated that the public’s trust in organizations responsible for GHs plays a crucial role in promoting and deploying the GB movement in China. Moreover, social trust had a greater influence on GH purchase intention than environmental concern, which implies that the effect of general environmental attitude is lower than that of specific social trust in the GH context.

### 5.2. Implications

#### 5.2.1. Theoretical Implications

This study provides some theoretical contributions. First, from an extended perspective of perceived value, this study develops a comprehensive research framework that incorporates residents’ perceptions and personal traits, enriching the literature on GH purchase intention. This study advocates that the perceptions of GHs’ attributes are more important than the attributes themselves in estimating the impact of GH purchase intention. This research perspective echoes calls for more attention to the impact of demand-side [12], which can help us have some insight into the potential causes for limited GH acceptance.

Second, we considered both residents’ positive and negative perceptions of GHs. Furthermore, this study attempted to make a thorough inquiry of the multi-dimensional nature of the perceptions of GHs, which has been ignored in previous literature. In more detail, perceived benefits were subdivided into four dimensions, namely, perceived functional benefits, perceived emotional benefits, perceived green benefits, and perceived social benefits, while perceived risks were subdivided into two dimensions, namely, perceived performance risks and perceived financial risks. Their relative effects on GH purchase intention were also examined. Thus, the study makes an important contribution to the new body of knowledge of the perceptions of GHs.

Finally, we explored the effect of personal psychological traits (environmental concern and social trust) on residents’ perception of GHs. Environmental concern is a widely examined personality factor in eco-innovation adoption research, and social trust is an important determinant of adopting a new product or technology in an early stage. We found that these two psychological traits have different impacts on residents’ perceptions and purchase intention of GHs. This finding provides insight into the effects of personal psychological traits on GH purchase intention.

#### 5.2.2. Practical Implications

Based on the empirical results, we present proposals for policymakers and developers to promote the popularization of GHs. First, this study demonstrated the positive impacts of all types of perceived benefits on the value perception of GHs. Therefore, measures should be taken to highlight the benefits of GHs to enhance residents’ sense of gain, honor, and happiness from living in GHs. In detail, governments and industries could issue certain reports to provide full and accurate information on the comfort, health, and money savings of GHs to residents. Unlike the professional terms in the evaluation standard system, the content of these documents should be easy to understand and in simple terms for residents. In this way, residents can recognize the functional benefits that constitute the main selling points of GHs. It is necessary to help residents become aware of the social attributes of GHs using appropriate means. For example, the government and developers could emphasize the positive environmental externalities of GHs in public places or housing purchase sites and increase the public visibility of GH appraisement labels. These measures could make residents realize that GHs help shape their self-image and enhance social recognition, which is especially important for environmentalists and prosocial-oriented people. Additionally, promoting GHs to the public requires not only enlightening them with reason but also moving them with affection. For example, governments and industries could establish GH experience centers to provide residents with opportunities to visit or live in GHs. The actual experiences might awaken and stimulate their positive emotional expectations for a better living environment and ecological environment. In the past, the information that governments and industries conveyed to residents was mainly on the contributions of GHs to environmental protection. This environmental positioning may resonate with residents to a certain extent, but this alone is insufficient to promote residents’ GH purchase behavior. Therefore, communication with residents should shift from environment-positioned information to value-positioned information. For governments, it is necessary to continuously improve the content of the evaluation standards to make them as close to the value needs of residents as possible. In the publicity and promotion of green housing, developers need to highlight the information of self-interest. Moreover, due to residents’ demand for source reliability, the advertising media chosen by developers should be one that is accessible to the public and has a high level of credibility.

Second, considering the negative effects of perceived performance risks on the perceived value of GHs, governments, and industries should take practical measures to control the potential risks of GHs effectively. For instance, governments should further improve relevant laws and regulations and strengthen the supervision of the GH market to mitigate residents’ anxiety about the potential risks. Due to the long-standing problem of poor operations of GHs in China, residents have concerns about the performance of GHs during the operating stage. Therefore, it is necessary to implement a life-cycle monitoring mechanism of GHs for the design, construction, and operating stages to ensure the safety and reliability of GHs effectively. In addition, governments could provide developers and property management companies with training courses and other forms of guidance to improve the overall operations of GHs. For developers, it is essential to change the development and operating mode of housing and continuously conduct product iteration and innovation so as to achieve excellent GH performance throughout the entire service life.

Finally, our findings indicate that personal psychological traits play vital roles in the formation of residents’ perception of GHs. Environmental concern positively influenced perceived green benefits, perceived emotional benefits, and perceived social benefits. Thus, it is necessary for governments and industries to launch publicity campaigns to emphasize the seriousness of the current environmental problems, especially delivering information concerning the pollution generated in the construction and operations of traditional housing. Social trust had a positive effect on perceived benefits but had a negative effect on perceived risk. Therefore, the importance of social trust in shaping residents’ perception of GHs should be noted. The key to enhancing the overall social trust of residents is to improve the information transparency of the GH market, which requires governments and industries to disclose the construction information and energy-saving information of GHs to the public. In addition, GHs certified by authoritative appraisement labels more easily bring psychological safety to residents, thereby increasing their sense of trust. Thus, governments should continually standardize the GH appraisement label system to guarantee the authority of the system. Additionally, establishing a participation mechanism for residents in the review process is also an effective way to increase the credibility of the rating system. For developers, it is necessary to closely follow the issuance and certification process of authoritative GH rating systems and standards and conduct timely certification for the developed GHs.

## 6. Conclusions

From an extended perspective of perceived value, this study developed a comprehensive research model to investigate the effects of perceived value, perceived benefits, perceived risks, environmental concern, and social trust on residents’ intention to purchase GHs. The findings suggest that all types of perceived benefits have a positive effect on perceived value, and perceived performance risks have a negative effect on perceived value. However, perceived financial risks have no significant effect on perceived value. Furthermore, as a result of the tradeoff between the benefits and the risks, perceived value has a strong positive influence on residents’ intention to purchase GHs. Residents with higher levels of environmental concern and social trust are likely to perceive more benefits and fewer risks when considering purchasing GHs.

The present study also has several limitations. First, our research was conducted in China. Therefore, considering the economic and cultural differences between different countries, prudence is needed when extending the conclusions of this study to other countries. It is significant to obtain data from other countries and examine the discrepancies. Second, this research collected data using online survey platforms, which might result in homogeneous samples. In subsequent studies, person-to-person sampling methods and online surveys could be applied simultaneously. Last, the dependent variable in our research model is residents’ intention to purchase GHs instead of actual behavior. Thus, future research may incorporate actual purchase behavior into the model.

## Figures and Tables

**Table 1:** Measurement items.

Construct	Item	Measurement
Purchase intention (PI)	PI1	Compared with the traditional housings, I would prefer to GHs.
	PI2	The next time I purchase a house, I would give priority to GHs.
	PI3	I would like to recommend friends to purchase GHs.
Perceived value (PV)	PV1	Compared to the sacrifice that I need to make, GHs are worthwhile.
	PV2	GHs are considered to be a good buy.
	PV3	Overall, GHs deliver me good value.
Perceived functional benefit (PFB)	PFB1	GHs are conducive to improve the residents’ living comfort at home.
	PFB2	GHs are beneficial to improve the residents’ health conditions.
	PFB3	GHs are useful to reduce household expenditures, such as water and electricity charges.
	PFB4	GHs are favorable to improve the residents’ quality of living.
Perceived emotional benefit (PEB)	PEB1	Living in GHs would be enjoyable.
	PEB2	Living in GHs would give me pleasure.
	PEB3	Living in GHs would make me feel relaxed.
	PEB4	Living in GHs would bring me a sense of harmony with nature.
Perceived green benefit (PGB)	PGB1	GHs contribute to the prevention of climate warming.
	PGB2	GHs contribute to the reduction in the carbon footprint.
	PGB3	GHs contribute to environmental protection.
	PGB4	GHs contribute to the reduction in environmental pollution.
	PGB5	GHs contribute to the reduction in consumption of natural resource.
Perceived social benefit (PSB)	PSB1	Living in GHs would improve the way I am perceived.
	PSB2	Living in GHs would gain me social approval.
	PSB3	Living in GHs would make a good impression on others.
	PSB4	Living in GHs would help me to feel acceptable to others.
Perceived performance risk (PPR)	PPR1	GHs may fall short of the level of benefits I expect.
	PPR2	GHs may not work satisfactorily due to a low level of operation and management.
	PPR3	GHs may not perform the functions that were described by the developer.
	PPR4	GHs may not perform well and cause problems in my life.
Perceived financial risk (PFR)	PFR1	I am concerned that GHs are too expensive to purchase.
	PFR2	I am concerned that GHs may have higher maintenance costs than traditional housing.
	PFR3	I am concerned that GHs may have higher repair costs than traditional housing.
	PFR4	I am concerned about suffering financial losses when purchasing and living in GHs.
Environmental concern (EC)	EC1	I am concerned about the environment.
	EC2	I am willing to make sacrifices to protect the environment.
	EC3	I am emotionally involved in environmental protection issues.
Social trust (ST)	ST1	I trust the quality of assessment standards for GHs developed by the official authorities.
	ST2	I trust the experts’ evaluation in the GHs assessment process.
	ST3	I trust the authenticity of application documents provided by the developers/investors/consultants.

**Table 2:** Demographic profile of participants.

Category		Number	Percentage (%)
Gender	Female	361	49.6
	Male	367	50.4
Age	18–29	241	33.1
	30–39	309	42.5
	40–49	131	18.0
	50–59	33	4.5
	60 and above	14	1.9
Education level	High school and below	56	7.7
	Junior college	118	16.2
	Bachelor	418	57.4
	Master’s degree or above	136	18.7
Household income per year (CNY 10,000)	Less than 10	135	18.6
	10–15	156	21.4
	15–25	193	26.5
	25–50	180	24.7
	More than 50	64	8.8

**Table 3:** Scale properties.

Construct	Items	Standard Loadings	Cronbach’s α	CR	AVE
Purchase intention (PI)	PI1	0.933	0.911	0.944	0.850
	PI2	0.923			
	PI3	0.909			
Perceived value (PV)	PV1	0.892	0.884	0.928	0.811
	PV2	0.904			
	PV3	0.906			
Perceived functional benefit (PFB)	PFB1	0.885	0.867	0.909	0.715
	PFB2	0.867			
	PFB3	0.819			
	PFB4	0.809			
Perceived emotional benefit (PEB)	PEB1	0.896	0.909	0.936	0.786
	PEB2	0.887			
	PEB3	0.891			
	PEB4	0.872			
Perceived green benefit (PGB)	PGB1	0.860	0.917	0.938	0.751
	PGB2	0.858			
	PGB3	0.880			
	PGB4	0.904			
	PGB5	0.829			
Perceived social benefit (PSB)	PSB1	0.886	0.927	0.948	0.821
	PSB2	0.920			
	PSB3	0.923			
	PSB4	0.892			
Perceived performance risk (PPR)	PPR1	0.882	0.927	0.947	0.818
	PPR2	0.923			
	PPR3	0.916			
	PPR4	0.897			
Perceived financial risk (PFR)	PFR1	0.890	0.925	0.946	0.815
	PFR2	0.919			
	PFR3	0.919			
	PFR4	0.882			
Environmental concern (EC)	EC1	0.867	0.847	0.907	0.765
	EC2	0.884			
	EC3	0.873			
Social trust (ST)	ST1	0.893	0.882	0.927	0.809
	ST2	0.927			
	ST3	0.878			

**Table 4:** Correlation coefficient matrix and square roots of average variances extracted (AVEs).

	PI	PV	PFB	PEB	PGB	PSB	PPR	PFR	EC	ST
PI	0.922									
PV	0.663	0.901								
PFB	0.471	0.493	0.846							
PEB	0.536	0.546	0.481	0.887						
PGB	0.437	0.475	0.473	0.543	0.867					
PSB	0.502	0.554	0.371	0.625	0.461	0.906				
PRR	−0.217	−0.251	0.006	−0.057	−0.061	−0.175	0.905			
PFR	−0.164	−0.144	0.101	0.079	0.037	−0.053	0.667	0.903		
EC	0.396	0.372	0.507	0.296	0.384	0.298	−0.065	0.041	0.875	
ST	0.519	0.565	0.512	0.508	0.461	0.480	−0.225	−0.120	0.311	0.899

Note: Diagonal elements are the square root of AVE, and the others are the correlation between constructs.

**Table 5:** Path analysis and significance verification of the structural models.

Hypotheses	Path	Path Coefficient	*t*-Value	*p*-Value	Hypothesis Supported
H1	PV→PI	0.664	18.530	0.000	Yes
H2a	PFB→PV	0.256	5.014	0.000	Yes
H2b	PEB→PV	0.198	3.603	0.000	Yes
H2c	PGB→PV	0.127	2.583	0.010	Yes
H2d	PSB→PV	0.249	5.460	0.000	Yes
H3a	PPR→PV	−0.130	2.641	0.008	Yes
H3b	PFR→PV	−0.090	1.572	0.116	No
H4a	EC→PEB	0.153	4.069	0.000	Yes
H4b	EC→PGB	0.266	5.658	0.000	Yes
H4c	EC→PSB	0.165	4.584	0.000	Yes
H4d	EC→PFR	0.087	1.917	0.055	No
H5a	ST→PFB	0.512	15.290	0.000	Yes
H5b	ST→PEB	0.460	11.904	0.000	Yes
H5c	ST→PGB	0.379	9.712	0.000	Yes
H5d	ST→PSB	0.429	11.340	0.000	Yes
H5e	ST→PPR	−0.225	5.793	0.000	Yes
H5f	ST→PFR	−0.147	3.945	0.000	Yes

**Table 6:** Total effects.

Construct	Total Standardized Effects on GH Purchase Intention
PV	0.664
PFB	0.170
PEB	0.131
PGB	0.084
PSB	0.165
PPR	−0.086
PFR	−0.060
EC	0.064
ST	0.278

## Data Availability

The data presented in this study are available on request from the first author.
